# Substrate-Oriented Nanorod Scaffolds in Polymer–Fullerene Bulk Heterojunction Solar Cells

**DOI:** 10.1002/cphc.201301104

**Published:** 2014-03-20

**Authors:** Yuta Ogawa, Matthew S White, Lina Sun, Markus C Scharber, Niyazi Serdar Sariciftci, Tsukasa Yoshida

**Affiliations:** [a] Research Center for Organic Electronics (ROEL), Yamagata University 4-3-16 Jonan, Yonezawa, Yamagata 992-8510 (Japan) E-mail: yoshidat@yz.yamagata-u.ac.jp; [b] Institute for Physical Chemistry/LIOS, Johannes Kepler University Altenbergerstraße 69, 4040 Linz (Austria); [c] Department of Applied Chemistry, Faculty of Science, Beijing University of Chemical Technology Beijing 100029 (China)

**Keywords:** hybrids, morphology, nanorods, organic, solar cells

## Abstract

The use of a p-type inorganic semiconductor to form a nanorod scaffold within a polymer-fullerene bulk heterojunction solar cell is reported. The performance of this cell is compared to those made of the commonly used n-type scaffold of ZnO, which has been reported many times in the literature. The scaffold is designed to improve charge-carrier collection by increased mobility in thicker samples. Observations show that generally the device performance shows a negative correlation to nanorod length. By using CuSCN as a p-type inorganic scaffold, a very similar trend is observed.

## 1. Introduction

Hybrid organic/inorganic nanosystems offer an interesting potential avenue for photovoltaics. The two material classes offer distinct advantages and disadvantages, with carrier mobility (low in organic semiconductors, about 10^−2^ cm^2^ V^−1^s^−1^ and high in inorganic semiconductors, about 10^2^ cm^2^ V^−1^s^−1^) being an important difference. In polymer–fullerene bulk heterojunctions (BHJ), the morphology must be precisely tuned on multiple scales, which range from 1–100 nm. The tools for controlling domains of this size are not currently sufficient, and the fabrication of highly efficient devices relies on the spontaneous self-organization of the morphology.[1, 2] Ideally, the optimal morphology will also be the lowest energy configuration so that molecular diffusion upon heating is not problematic; this is possible, but it is by no means trivial.

Some inorganic semiconductors have properties that could be very beneficial in organic BHJ solar cells. There are many such materials that can be grown into nanostructures of various shapes and sizes, which are then crystalline and stable. These nanostructures can be free particles (dots, rods, tetrapods, etc.) or can be grown in an oriented manner from a device-relevant substrate, such as ITO.[3–7] These inorganic scaffolds allow for a predetermined and stable morphology, typically with very high carrier mobility, which gives more control and more freedom in device design, and as such the topic has been the subject of intense research.[8]

Organic semiconductors generate excitons upon photoexcitation, and therefore require a heterojunction to separate into free carriers. The most successful heterojunctions have been polymer–fullerene combinations.[9–12] The most obvious step to introduce the inorganic nanostructures into organic photovoltaics (OPV) is to replace the fullerene in the heterojunction with an n-type semiconductor such as ZnO. This approach has been studied extensively with reasonable success, however the resulting devices are not particularly efficient compared to those with fullerenes.[4, 13–17] For example, semiconducting polymers intermixed into a ZnO nanorod scaffold can produce devices ranging from 0.5 %–1.6 % power conversion efficiency,[17, 18] whereas polymers with fullerene produce 4 %–9 %.[10, 11, 19, 20] This lower efficiency is mostly due to insufficient interfacial area between the two phases, which leads to low short-circuit currents. If you create a nanostructure with fine enough pores, it is nearly impossible to put the organic semiconductor into it, and therefore the resulting domain size remains too large to efficiently separate excitons.[21]

The next logical step is to incorporate an inorganic scaffold into a polymer–fullerene BHJ. Here the polymer–fullerene domains are small, and the inorganic scaffold can act as a high-mobility continuous charge-collection pathway direct to the desired electrode for one selected carrier. The carrier mobility in organic semiconductors is low and considered a limiting factor in solar cells. The nanorod scaffold could allow for much thicker solar cells with high short-circuit current (*J*_SC_) and fill factor (FF) values. This method is reasonably successful, in that it improves the short circuit current significantly (compared to a polymer–ZnO nanostructured heterojunction), roughly to the same value you would expect from a polymer–fullerene BHJ. This type of solar cell has been studied by using a variety of inorganic and organic donor/acceptor material combinations with successful, though not necessarily conclusive results.[16, 17, 22–28]

Herein we attempt to answer the question: Does a substrate-oriented nanorod scaffold improve the functionality of polymer–fullerene BHJ solar cells? Specifically we compare the published efficiency values of the most common material combinations (ZnO scaffold with poly(3-hexylthiophene):phenyl C_61_-butryric acid methyl ester (P3HT:PCBM) and Ag, or poly(3,4-ethylenedioxythiophene):poly(styrenesulfonate) (PEDOT:PSS)/Ag. The trend appears to be a quite general decrease in efficiency with nanorod length. Based on this analysis we suppose that the electron mobility (which is enhanced by ZnO nanostructure) may not be a limiting factor in device efficiency. A nearly identical nanostructure may be constructed from the hole-transporting CuSCN. We present a series of solar cells using CuSCN scaffold and P3HT:PCBM BHJ with a Ca/Al electron-selective electrode. These devices have the same nanostructure and the same active layer, but should exhibit enhanced hole transport, compared to the enhanced electron transport in the ZnO. We vary the length of the CuSCN nanorods from 0–800 nm to compare the trend in performance for devices with the same nanostructure and active layer, but with selectively “enhanced” electron (ZnO) and hole (CuSCN) transport. The trend is the same for both types of devices, in that solar cells on the nanorod scaffolds are functional, but the efficiency appears to decrease with rod length continuously from 0 nm.

## 2. Results and Discussion

### 2.1. ZnO-Scaffold Solar Cells

The discussion of inorganic scaffolds begins with ZnO, which is the most common inorganic material used in nanorod-scaffold hybrid solar cells, for several reasons.[8] ZnO is an n-type semiconductor with a conduction-band energy level that is well aligned to the LUMO of PCBM, and it is known to function nicely as an electron-selective contact in BHJ solar cells;[5–7, 31–38] it is transparent in the visible and IR portions of the solar spectrum, and is nontoxic, abundant, and inexpensive. Perhaps the strongest motivation for using ZnO in particular is that various physical and chemical methods may be used to grow nanostructures of almost innumerable shapes, sizes, and aspect ratios, which allows for a high degree of morphological control when designing solar cells.[1, 2, 5–7, 31–38]

In 2003, Vayssieres showed that arrayed nanorods of ZnO may be grown, oriented roughly perpendicular to a substrate, from aqueous solution.[7] The nanorods can be grown at large scales and with low costs.[6] More importantly, the length and aspect ratio of the rods can be controlled by growth time and concentration, and the orientation (perpendicular to the substrate or random orientation) is controlled by the quality of the seed layer. The length can be varied from several tens of nanometers to several micrometers, and the width from tens to hundreds of nanometers, and the spacing can be controlled on the order of tens of nanometers. These are precisely the approximate length scales that are applicable in nanorod-scaffold hybrid PV.

**Figure 1:**
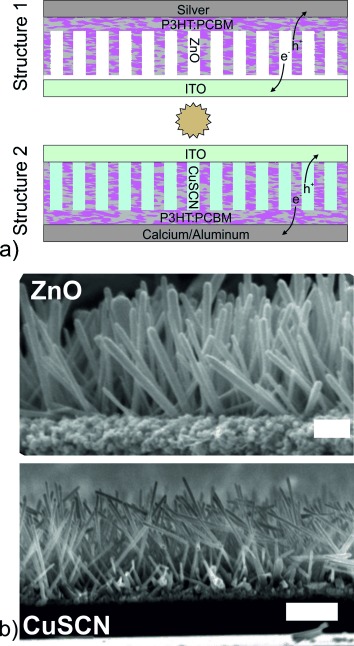
a) Drawings of the nanorod-scaffold-based BHJ solar cells with Structure 1 consisting of ITO/ZnO/P3HT:PCBM/Ag or PEDOT:PSS/Ag in an inverted cell, and Structure 2 consisting of ITO/CuSCN/P3HT:PCBM/Ca:Al. b) Cross-sectional SEM images of ZnO nanorods (adapted with permission),[7] and CuSCN nanorods, with scale bars representing 1 μm in both images.

Because the energetic and morphological properties of this ZnO scaffold were an ideal test bed for hybrid PV, research efforts to incorporate ZnO nanorods into polymer solar cells intensified worldwide. In 2006, Olson et al. showed that the ZnO scaffold functioned as a heterojunction-acceptor material with P3HT polymer, and that the scaffold can be used as an electron-selective contact in a P3HT:PCBM BHJ solar cell with an Ag electrode.[17, 29] This was the first report of a device with Structure 1 (see Figure 1), and showed 2 % PCE with roughly 150–200-nm-long nanorods. Subsequently they published the same device architecture but with only the ZnO seed layer, and no nanorods at all, with device efficiency of 3 %.[29]

Within the first year of the nanorod-scaffold polymer–fullerene BHJ solar cell, there was already evidence that the nanorods were detrimental to device performance. However, it was difficult to conclude anything from these two data points. There has since been extensive research on precisely this device structure.

**Figure 2:**
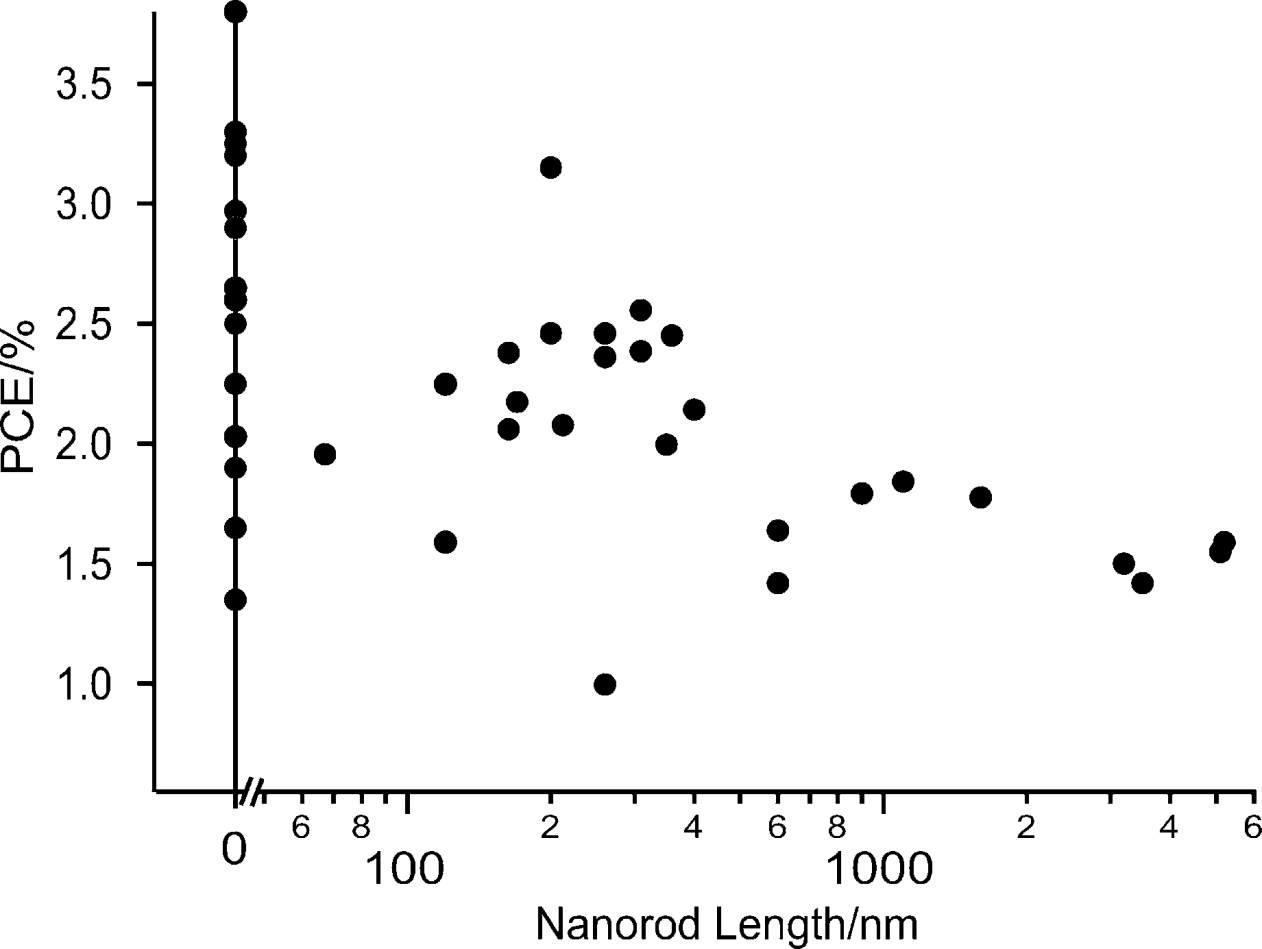
Power conversion efficiency versus ZnO nanorod length in devices constructed using Structure 1. Data was compiled from reported literature values.[17, 23–29, 39]

In Figure 2 we plot the reported efficiency of the solar cells as a function of ZnO nanorod length for over 40 reported cells using similar structures.[17, 23–29, 39] We collected the information for cells with only the materials described in Structure 1: ZnO-nanorod scaffold, P3HT:PCBM BHJ blend, and either Ag or PEDOT:PSS/Ag electrode. Other materials that have been used, including different semiconducting polymers, oxide scaffolds, and hole-selective contacts, but these reports are more isolated. The materials identified in Structure 1 are used commonly enough to provide some comparison across multiple studies and multiple laboratories. We also compare many of the reported values that use the same materials but with no scaffold at all.

In polymer-photovoltaics research, there is a high degree of variation from batch-to-batch, and from one laboratory to another, due to subtle processing differences. To produce a high efficiency cell requires an intense optimization procedure for each variable. The ZnO nanorods themselves, if uniform stoichiometric composition is assumed, have variables of length, aspect ratio, spacing, orientation, and seed-layer thickness. Other variables include the quality of P3HT available (which has improved over time), and the tools and environment used for device fabrication and characterization. It is not at all surprising that the data in Figure 2 show such dispersion.

Of the individual studies published in the literature, there are specific results that stand out. In one study, the PCE of the cells was observed to increase with rod length while maintaining constant *J*_SC_.[28] In other studies, the PCE of the cells was observed to decrease.[17, 23, 29] Given that there are significant opportunities for errors in the efficiency measurements, and that the optimization of each cell is extremely labor-intensive, we felt that a blind comparison of the reported PCEs was the best way to check for a global trend.

The comparison in Figure 2 shows a decreasing PCE with increasing nanorod length. Because all of these data represent devices that were independently optimized to the point of being considered of publication quality, they represent a good statistical cross-section of what can be expected from the material combination. The cumulative data suggest that such ZnO scaffolds are not yet demonstrably beneficial to the PCE of BHJ solar cells.

Within the P3HT:PCBM BHJ blend, the electron and hole mobilities can vary by up to an order of magnitude, depending on the ratio of the two materials and the BHJ nanoscale morphology. However, the electron mobility is typically found to be slightly higher than the hole mobility, by up to a factor of 2. So perhaps it is the case that the ZnO scaffold (mobility presumably greater than 10 cm^2^ V^−1^s^−1^)[40] selectively enhances the transport of the faster carrier.

### 2.2. CuSCN-Scaffold Solar Cells

If a similar, theoretically “ideal”, nanorod scaffold could be applied to enhance the hole transport in the same polymer–fullerene blend, would it show overall efficiency enhancement? The first challenge in answering this question is to identify an appropriate inorganic semiconductor. CuSCN is a p-type material (with a hole mobility presumably greater than 0.1 cm^2^ V^−1^s^−1^)[41] that has been demonstrated to function as a hole-selective contact for OPV. Takahashi et al. demonstrated solar cells with ITO/CuSCN/P3HT:PCBM/Al structure and 2.5 % PCE.[42] In 2011, Sun et al. demonstrated that arrayed nanorods of CuSCN can be electrodeposited from solution onto conducting substrates.[43] The nanostructure is nearly identical to the ZnO scaffold used extensively in solar-cell research, as can be seen in Figure 1 b. These CuSCN nanorod scaffolds will provide a “hole-mobility-enhancing” test structure for solar cells.

**Figure 3:**
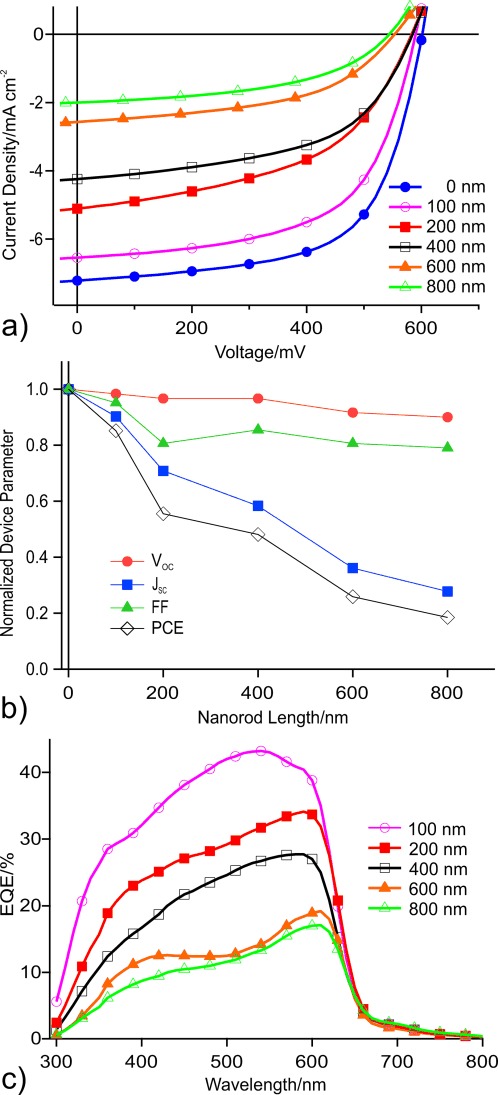
a) *J*–*V* characteristics of solar cells constructed using Structure 2, for various CuSCN nanorod lengths. b) Normalized device parameters versus CuSCN nanorod length. c) EQE curve for devices of different nanorod lengths.

In Figure 3 a we plot the *J*–*V* (current density–potential) characteristics of the solar cells for rod lengths varying from 0 (no CuSCN) to 800 nm. The observed trend in PCE is very similar to that seen in the literature values of devices with ZnO scaffolds. CuSCN is demonstrated to function as a hole-transport layer and the nanorod scaffold is successfully introduced into functional solar cells. However increasing nanorod length makes the devices progressively worse.

The device parameters (*V*_OC_, *J*_SC_, FF, PCE, serial resistance, and parallel resistance, *V*_OC_=open circuit voltage), are reported in Table 1. The values, normalized to the 0 nm rod-length device, are plotted in Figure 3 b. The value of *V*_OC_ is relatively constant while the values of FF and *J*_SC_ both decrease with rod length. It is not surprising that FF decreases with increased device thickness, due to increased recombination and higher likelihood of protruding nanorods causing shorts. To understand the decrease in *J*_SC_, we look to the EQE data shown in Figure 3 c. We see a persistent decrease in the EQE intensity, as well as a change in shape of the spectrum. The higher energy light contributes proportionally less to the photocurrent in thicker scaffolds. This result is likely due to the optical absorption of CuSCN, which is strong for wavelengths shorter than 350 nm.

**Table 1:** Device parameters for CuSCN-based solar cells of various nanorod lengths. The 0 nm device has no CuSCN at all

Rod length [nm]	*V*_OC_ [mV]	*J*_SC_ [mA cm^−2^]	FF [%]	PCE [%]	R series [Ω]	R parallel [kΩ]
0	600	7.2	62	2.7	40	110
100	590	6.5	59	2.3	50	270
200	580	5.1	50	1.5	130	160
400	580	4.2	53	1.3	100	170
600	550	2.6	50	0.7	200	190
800	540	2	49	0.5	40	50

The purpose of introducing a nanorod scaffold is to improve the values of *J*_SC_ and FF by enhancing carrier transport and preventing recombination, but these are precisely the parameters that decrease sharply with rod length. This was not the case for the ZnO-scaffold devices in the literature, where the device parameter trends were far less consistent. However, it is a reasonable indication that the structure does not perform the desired function of improving the solar-cell performance.

A very recent report by Chappaz-Gillot et al. demonstrates two similar CuSCN structures in conjunction with a low-bandgap semiconducting polymer and a TiO_*x*_ hole-blocking layer to form scaffold-supported BHJ solar cells.[44] The authors present several devices with nanocolumnar CuSCN of varying thickness, which show the same trend in PCE as is observed here.

### 2.3. Analysis

In both the ZnO and CuSCN scaffold devices, we observe a general behavior for which there are several possible explanations. Primarily, the real scaffold-based solar cells do not conform to the structures shown in Figure 1 a. There are many variables involved in such a device, including the rod length, aspect-ratio, spacing, and orientation with respect to the substrate, as well as the degree to which the BHJ blend infiltrates the scaffold and the thickness of the organic capping layer that separates the scaffold from the metal electrode. The capping layer is necessary to prevent shorting of the metal directly to the scaffold, which would reduce the parallel resistance of the solar cell. If the capping layer is too thick, it adds significant series resistance. Full infiltration of the BHJ blend would be ideal, but is certainly not guaranteed. For densely packed, vertically oriented nanorods, polymers will typically sit on top of the scaffold. Almost universally, the nanorod-scaffold-based devices are constructed on highly disordered nanorods as seen in Figure 1 b, which allow for much better filling of the BHJ blend material. In the case of some of the longer ZnO nanorods, the BHJ blend tends to coat the nanorods without filling the voids, which requires a space-filling PEDOT:PSS layer or conformal coating of the metal electrode.[23, 25]

In our CuSCN devices, we do not see any conclusive evidence of either nanorod protrusion, excessive capping layer, or failure of the BHJ blend to penetrate the scaffold. A protruding nanorod scaffold would be in direct contact with both the ITO and the metal electrode and thus provide decrease the parallel resistance of the diodes. Likewise, overly thick capping layers would add significant serial resistance. From the data presented in Table 1, we observe no corresponding trend with rod length. We can also see in cross-sectional SEM images (Figure 4 S) that there is sufficient capping of the rods with the BHJ blend. The BHJ blend seems to infiltrate the gap between the CuSCN nanorods rather well, although the absolute (quantitative) degree of infiltration cannot be estimated from these images.

There may also be more fundamental problems with the proposed geometry. Following the dissociation of the exciton in the BHJ blend, either a built-in field or a concentration gradient are required to induce drift or diffusion current in any BHJ solar cell. The selective contact materials are responsible for this requirement. In the device architecture proposed here (Figure 1 a), the BHJ blend material deepest within the scaffold is primarily responsible for light absorption as it is closest to the transparent electrode. In these regions, the carrier concentration gradient that is generated by charge transfer through the walls of the nanorod scaffold is directly perpendicular to the desired direction of current flow. The presence of the scaffold with high conductivity and dielectric constant (compared to the BHJ layer) will alter and perhaps completely shield the field within the scaffold. If the scaffold is significantly more conductive than the BHJ blend, then the field will be concentrated in the region between the tips of the nanorods and the metal back electrode. While the scaffold may selectively enhance the extraction of one carrier, it may also diminish the extraction of the other and leave behind an excess of one carrier in the region of primary photon absorption.

## 3. Conclusions

The inorganic nanorod scaffold, incorporated into polymer–fullerene BHJ solar cells, is an interesting approach to merge two complementary nanotechnologies. The motivation for using this system is to potentially improve carrier transport through direct, carrier-selective, connected pathways. Unfortunately, we do not see any consistent improvement in solar-cell performance. Devices without nanorods have higher power conversion efficiency, on average, and peak reported values than those without. There is more evidence for a negative correlation between rod length and device performance.

This trend appears to be true for both electron-transport and hole-transport nanorod scaffolds. We analyzed nearly a decade of published results of the ZnO/P3HT:PCBM material combination. While several of the individual studies see positive effects of the nanorod scaffold, this is not a universal observation. Furthermore, the overall picture presented by the multiple independent results is of decreasing PCE with increasing rod length.

We constructed a nearly identical nanorod scaffold with the p-type semiconductor CuSCN. By using it in the P3HT:PCBM BHJ solar cell in a comparable configuration, we see a similar negative correlation. The poor performance of the solar cells is mostly due to decreasing values of *J*_SC_ and FF, which are precisely the areas that we hoped to improve with the scaffold.

This hybrid solar cell design has been investigated for a decade, following the introduction of the arrayed nanorods of ZnO. In that time, we have seen the PCE of conventional polymer–fullerene-BHJ solar cells improve to over 9 %.[11] Without the nanorods, the devices have demonstrated FF values of 79 %[45] and *J*_SC_ over 17 mA cm^−2^.[11]

## Experimental Section

### Electrodeposition of CuSCN Nanorods

Highly oriented CuSCN nanorods were obtained by electrodeposition according to the method described in the literature.[43] An ITO (10 Ω sq.^−1^, Lumtec) coated glass (20×25×0.7 mm) patterned for four cells was used as the substrate. Masking tape was applied to conductive pads of 8.5×3.0 mm to regulate the exposed area to 3.5 ×3.0 mm. Since four such pads we used in parallel, the effective electrode area was about 0.42 cm^2^. Potentiostatic electrolysis at +0.2 V (vs. Ag/AgCl) was carried out for a controlled period between 2–20 min at room temperature in an ethanol/water mixed solution (1:1 in volume ratio) containing 10 mm Cu(ClO_4_)_2_ (Aldrich), 5 mm LiSCN (Kishida), and 0.1 m LiClO_4_ (Wako). The electrode was in a stationary condition and no agitation of the solution was applied, so that the reactants were depleted in the vicinity of the electrode to enhance the anisotropic growth of the nanorods.[43] The electrodeposited CuSCN was rinsed with water and dried in air at room temperature. The nanorod length was determined by observing the cross-section with a scanning electron microscope (JEOL JSM-6700F), while the crystallographic orientation of the nanorod was checked by measuring X-ray diffraction (XRD) pattern on a Rigaku SmartLab. Additional details of the CuSCN nanorod growth and characterization can be found online in Supporting Information Figures S1–S3.

### Fabrication of BHJ Cell and Measurements

BHJ layers were prepared either on bare ITO or on CuSCN-nanorod layer by spin-coating from an *o*-dichlorobenzene (DCB) solution containing poly-3-hexylthiophene (P3HT, Lumtec) and [6,6]-phenyl-C_61_-butyric acid methyl ester (PCBM, Lumtec) in equal weight (each 20 mg per 1 mL DCB) at a rate of 500 rpm for 28 s and then at 1000 rpm for 2 s, as it was found to be suitable to fill up the nano-space of CuSCN scaffolds with the BHJ material. The samples were kept in a glove-box filled with N_2_ and left for 2 h for slowly drying the BHJ layer and finally were annealed at 110 °C on a hotplate. The samples were then transferred to a vacuum chamber for depositing 20-nm thick Ca and 100-nm thick Al layers in strips of 3.0-mm width to be overlaid on the BHJ-coated CuSCN layer to construct four cells of 3.0×3.0 mm per substrate. The active part was then protected by a glass cap filled with desiccant before the cell was taken out of the glove-box.

The *I*–*V* curve and the photocurrent action spectrum of the cell were measured on a Bunko Keiki CEP-2000 system. While the *I*–*V* curve was measured under illumination with an AM 1.5 simulated sun light (100 mW cm^−2^), the external quantum efficiency (EQE) was measured under monochromatic light illumination with a constant photon flux (5.0×10^15^ s^−1^ cm^−2^).

## Supporting Information

As a service to our authors and readers, this journal provides supporting information supplied by the authors. Such materials are peer reviewed and may be re-organized for online delivery, but are not copy-edited or typeset. Technical support issues arising from supporting information (other than missing files) should be addressed to the authors.

miscellaneous_information
