# Supplementary material for: Substrate-Oriented Nanorod Scaffolds in Polymer–Fullerene Bulk Heterojunction Solar Cells
Source: Chemphyschem. 2014 Mar 20;15(6):1070–5. doi: 10.1002/cphc.201301104 (PMC4501325; doi:10.1002/cphc.201301104)
Supplement: Supplementary file 1 — miscellaneous_information [file cphc0015-1070-sd1.pdf]

CHEMPHYSCHEM

Supporting Information

© Copyright Wiley-VCH Verlag GmbH & Co. KGaA, 69451 Weinheim, 2014

Substrate-Oriented Nanorod Scaffolds in Polymer–Fullerene Bulk Heterojunction Solar Cells

Yuta Ogawa,[a] Matthew S. White,[b] Lina Sun,[a, c] Markus C. Scharber,[b]
Niyazi Serdar Sariciftci,[b] and Tsukasa Yoshida*[a]

cphc_201301104_sm_miscellaneous_information.pdf

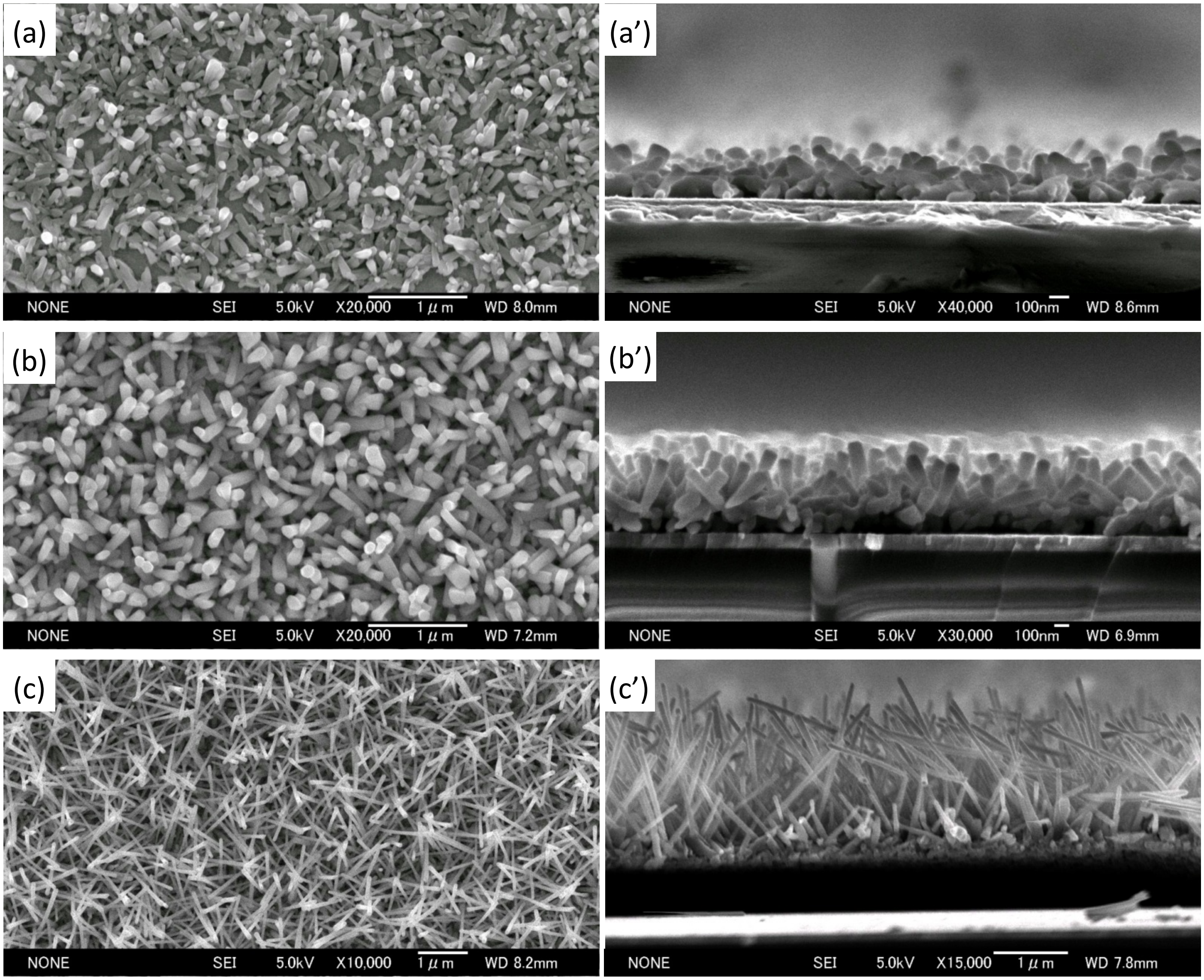

Fig. 1S SEM pictures of CuSCN nanorods electrodeposited on ITO glass electrodes for 5 (a, a’), 20 (b, b’) and 60 (c, c’) min. at +0.2 V (vs. Ag/AgCl) from ethanol / water mixed solution (1 /1 in volume ratio) containing 10 mM $Cu(ClO_4)_2$, 5 mM LiSCN and 0.1 M $LiClO_4$. It appears that the most of the nanorods are laid down in the beginning of the electrolysis. Those oriented with the longitudinal side vertical to the substrate plane continue to grow, while those erroneously oriented are buried underneath, to make vertically aligned nanorod structure eventually.

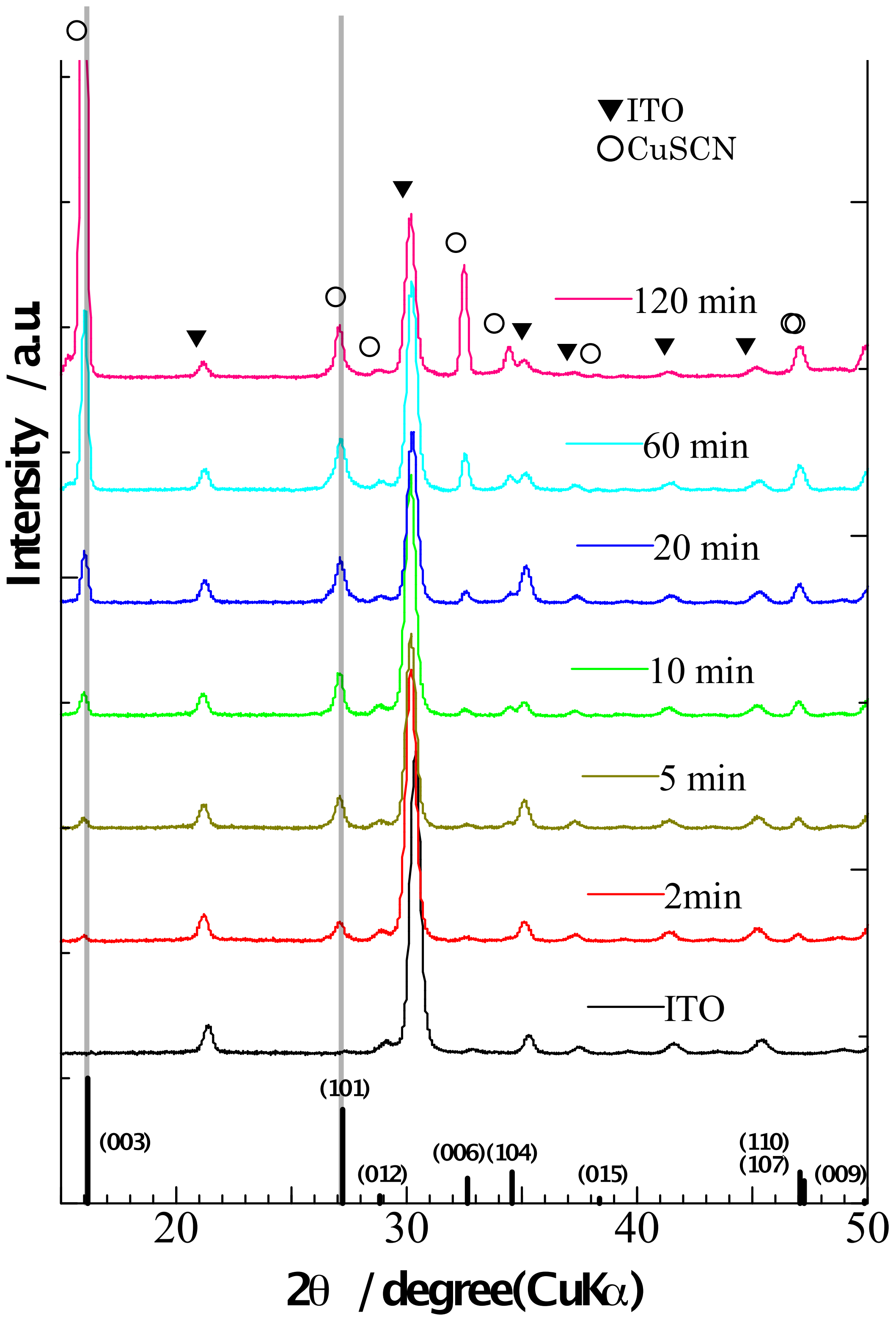

Fig. 2S XRD patterns of CuSCN nanorods electrodeposited on ITO glass electrodes for 2, 5, 10, 20, 60 and 120 min. at +0.2 V (vs. Ag/AgCl) from ethanol / water mixed solution (1 /1 in volume ratio) containing 10 mM $Cu(ClO_4)_2$, 5 mM LiSCN and 0.1 M $LiClO_4$ in comparison with that of a bare ITO and powder diffraction standard of β-CuSCN (JCPDS 29-0581). One can notice changes in the relative intensity of the two major diffraction peaks from the (003) and (101) planes marked with the grey lines. Quantitative treatment of the peak intensity shown in Fig. 3S reveals changes of the crystallographic orientation along the growth of the CuSCN nanorods.

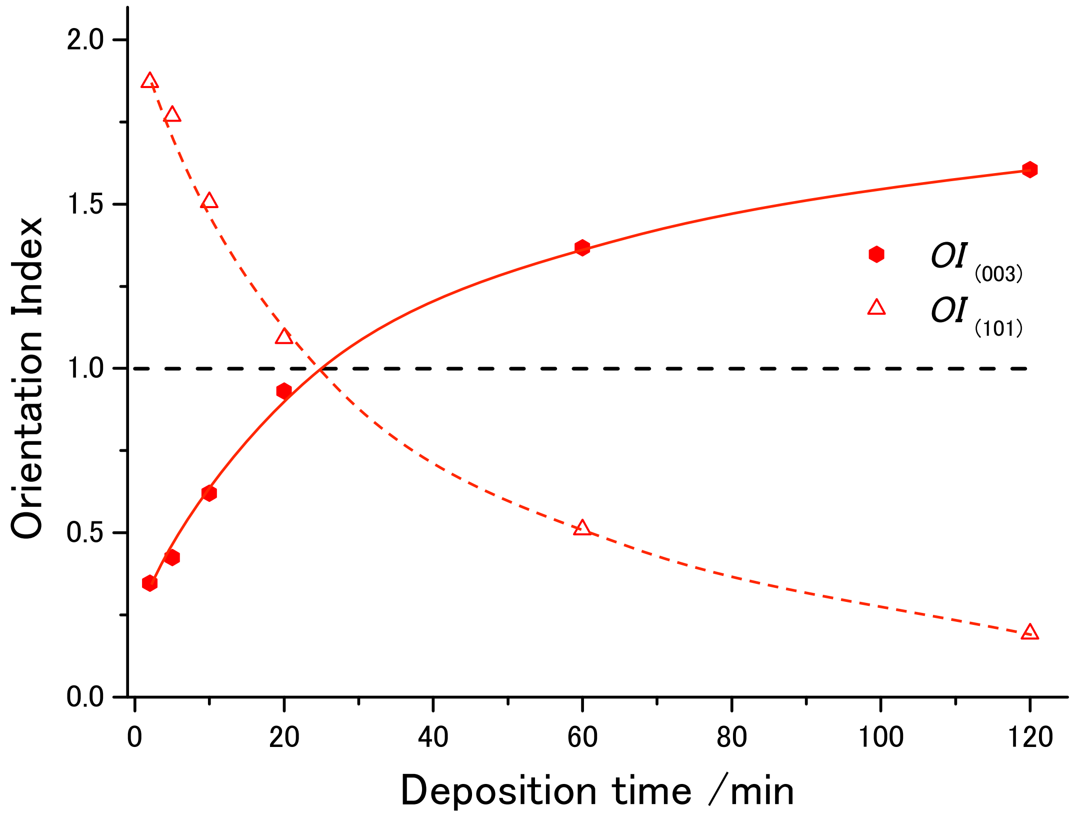

Fig. 3S Change of orientation indices ($OI_{(003)}$ and $OI_{(101)}$) determined from the XRD patterns in Fig. 2S. In the beginning, the crystallites are oriented to lay down the *c*-axis as recognized from the high $OI_{(101)}$ value. As the nanorod grows, the $OI_{(003)}$ value increases with the expense of the $OI_{(101)}$, indicating the preferential orientation to align the *c*-axis vertical to the substrate plane. Comparison to the morphological change shown in Fig. 1S reveals that the longitudinal side of the CuSCN nanorod corresponds to the *c*-axis of β-CuSCN.

<u>The method for calculation of orientation indices</u>

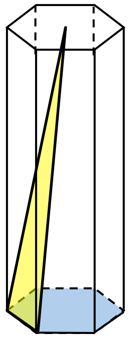

β-CuSCN
a=3.857Å
c=16.449Å

(003)∠(101) = 78.5°

(003) and (101) are nearly perpendicular

β-CuSCN has an elongated hexagonal unit cell. Comparison of the peak intensities from the (003) and (110) planes is a good measure of the orientation of the crystallites, since these planes are perpendicular from each other. However the (110) diffraction peak overlaps with that of the (107) planes which are nearly parallel with the (003) (See Fig. 2S). Therefore, the (003) peak intensity was compared to that of the (101) planes which are close to parallel with the *c*-axis.

The orientation indices (*OI*) were defined as follows. The intensity factors of the film samples (IF_f) were calculated from the measured peak intensities, and were divided by those of the powder standard (IF_p).

$$IF_{p(003)}=\frac{I_{p(003)}}{I_{p(003)}+I_{p(101)}}=\frac{100}{100+75}=0.571$$

$$IF_{p(101)}=\frac{I_{p(101)}}{I_{p(003)}+I_{p(101)}}=\frac{75}{100+75}=0.429$$

$$IF_{f(003)}=\frac{I_{f(003)}}{I_{f(003)}+I_{f(101)}} \qquad IF_{f(101)}=\frac{I_{f(101)}}{I_{f(003)}+I_{f(101)}}$$

$$OI_{(003)}=\frac{IF_{f(003)}}{IF_{p(003)}} \qquad OI_{(101)}=\frac{IF_{f(101)}}{IF_{p(101)}}$$

When $OI_{(hkl)} > 1$, the planes are preferentially oriented in parallel with the substrate plane.

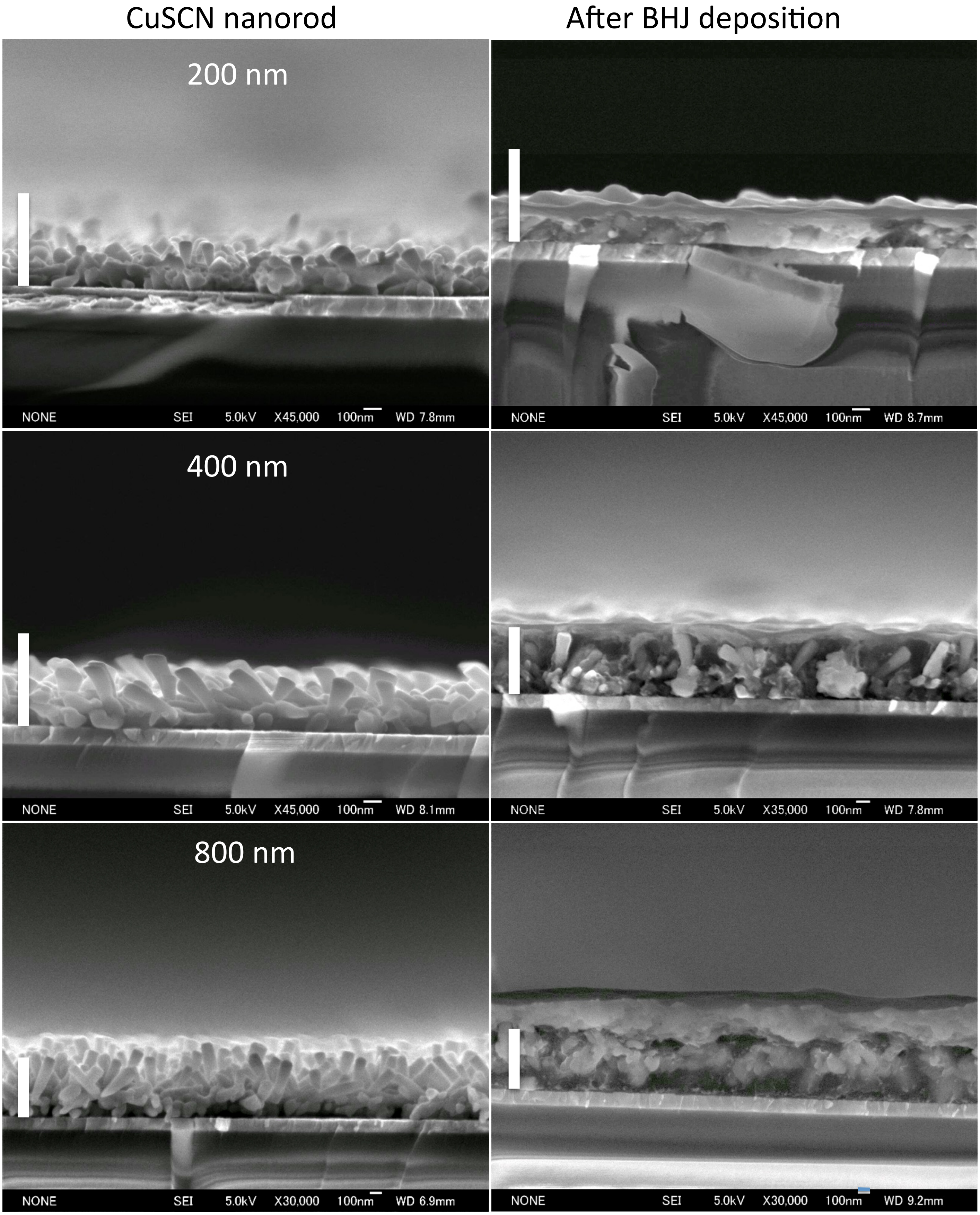

Figure 4S. Cross-sectional SEM images of 200 nm, 400 nm, and 800 nm CuSCN scaffolds without, and with BHJ layer. Images were taken at different magnifications (labeled) and a vertical white scale bar representing 500 nm was added to the left edge of each image aligned with the surface of the ITO. Polymer seems to be infiltrating the gap between CuSCN nanorods rather well, even though the absolute (quantitative) degree of infiltration cannot be estimated from these images. Protrusion of CuSCN rods to short the device are not likely to be a problem, as the top of the rods are well covered with the polymer blend.
